# Predictive Assessment of the Antiviral Properties of *Imperata cylindrica* against SARS-CoV-2

**DOI:** 10.1155/2024/8598708

**Published:** 2024-08-04

**Authors:** Frank Eric Tatsing Foka, Hazel Tumelo Mufhandu

**Affiliations:** Department of Microbiology Virology Laboratory School of Biological Sciences Faculty of Natural and Agricultural Sciences North West University, Mafikeng, Private Bag X2046, Mmabatho, South Africa

## Abstract

The omicron variant and its sublineages are highly contagious, and they still constitute a global source of concern despite vaccinations. Hospitalizations and mortality rates resulting from infections by these variants of concern are still common. The existing therapeutic alternatives have presented various setbacks such as low potency, poor pharmacokinetic profiles, and drug resistance. The need for alternative therapeutic options cannot be overemphasized. Plants and their phytochemicals present interesting characteristics that make them suitable candidates for the development of antiviral therapeutic agents. This study aimed to investigate the antiviral potential of *Imperata cylindrica* (*I. cylindrica*). Specifically, the objective of this study was to identify *I. cylindrica* phytochemicals that display inhibitory effects against SARS-CoV-2 main protease (M^pro^), a highly conserved protein among coronaviruses. Molecular docking and *in silico* pharmacokinetic assays were used to assess 72 phytocompounds that are found in *I. cylindrica* as ligands and M^pro^ (6LU7) as the target. Only eight phytochemicals (bifendate, cylindrene, tabanone, siderin, 5-hydroxy-2-[2-(2-hydroxyphenyl)ethyl]-4H-1-benzopyran-4-one, maritimin, 5-methoxyflavone, and flavone) displayed high binding affinities with M^pro^ with docking scores ranging from −5.6 kcal/mol to −9.1 kcal/mol. The *in silico* pharmacokinetic and toxicological assays revealed that tabanone was the best and safest phytochemical for the development of an inhibitory agent against coronavirus main protease. Thus, the study served as a baseline for further *in vitro* and *in vivo* assessment of this phytochemical against M^pro^ of SARS-CoV-2 variants of concern to validate these *in silico* findings.

## 1. Introduction

**Figure 1:**
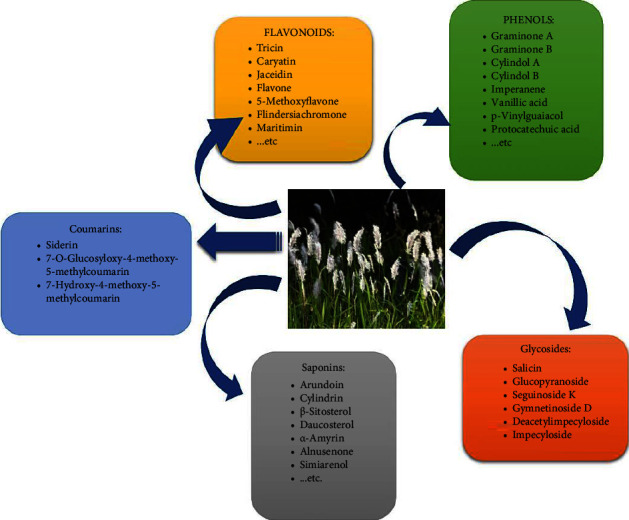
*Imperata cylindrica* and its phytochemicals.

Commonly known as “speargrass,” “cogongrass,” “alang-alang” or “kunai grass,” *Imperata cylindrica* is a member of the Poaceae family [1]. It is a perennial rhizomatous plant that can grow on dry soils or soils with high moisture content to reach 0.6–3 m height. The roots of the plant are fibrous and can thrive 1 to 1.2 m deep in the soil. The leaves are stiff, measuring up to 120 cm long and 2 cm wide at the base with a narrowing to sharp point at the top and a prominent white midrib. The flowers are white fluffy spike-like heads of 5–20 cm long and 2.5 cm diameter. The plant produces brown, oblong seeds that are 1–1.5 mm long, with a ring of silky white hairs that are 10 mm long around the base (Figure 1) [2]. It is native and widely found in tropical and subtropical Asia, Oceania, Australia, Southern Europe, and Eastern Africa [3]. It was inadvertently introduced in Latin America, the Caribbean, and the south-eastern United States through contaminated shipping packages and intentionally as an ornamental plant and as forage grass for erosion control [4]. As a result of its pyrophytic nature, it has been associated with wildfires in areas that it colonizes and is often regarded as an invasive weed which naturalises aggressively through self-seeding [5]. In such cases, it forms a monoculture that displaces native species, leading to low crop yield, low crop quality, reduction of farm size, increased labour requirement, and increased susceptibility to crop pathogens [5, 6].

Despite these negative aspects, *I. cylindrica* extracts have numerous beneficial attributes that justify its usage as a traditional medicine to treat various ailments [1, 3, 7, 8]. For example, in the traditional system of the “Shen Nong's herbal Classic” of the Han Dynasty (China), the plant's medicinal benefits are attributed to the lung, stomach, and bladder meridian and it is also acclaimed for its antidiuretic effects and fever-relieving properties [1]. Moreover, it is indicated in the replenishment of the spleen, the treatment of polydipsia, blood stasis, lung heat, difficulty in micturition, irregular menses, edema, and jaundice [8]. About 72 phytochemicals have been isolated from *I. cylindrica* and characterized extensively. The main types of phytochemicals that have been characterized belong to the classes of flavonoids, saponins, coumarins, glycosides, and phenols. A few compounds of each class of phytochemicals are shown in Figure 1 [7, 9–13]. Other unclassified compounds such as sesquiterpenoid, cylindrene, tabanone, palmitic acid, and phytol were also isolated from this plant. It has shown that *I. cylindrica* extracts which contain these compounds display various biological activities. For instance, phytol, palmitic acid, and coumarin were proven to exhibit antibacterial activities [1, 14], while imperanene (a phenol) and chromone (a flavonoid) exhibit platelet aggregation activity and neuroprotective properties, respectively [15, 16]. Other biological activities of *I. cylindrica* extracts are hepatoprotective activities [17], vasodilative properties [18], antioxidant properties [19], anti-cancer activities [19–21], and anti-inflammatory activities [1]. Moreover, the rhizome of this plant can be used alone or in combination with other medicinal plants such as *Panax quinquefolius*, *Rehmannia glutinosa*, and *Wolfiporia cocos* to treat hematuresis [1]. In addition, it is also used in combination with *Artemisia capillaris* Thunb and *Gardenia jasminoides* Ellis to reduce dampness and heat in the management of jaundice. Furthermore, *I. cylindrica* is used together with *Morus alba* L. and *Puerariae lobatae* Radix for the treatment of asthma and high fever, respectively [1].

SARS-CoV-2 emerged as a life-threatening virus in 2019 and claimed millions of life worldwide. With the continued emergence of variants of concern, coupled with the inefficacy of the initial drugs and a rise in the number of hospitalizations and deaths, the call for an effective vaccine became urgent [22]. Precisely, the omicron variant of SARS-CoV-2 (B.1.1.529), classified as a variant of concern (VOC), has swiftly propagated across the globe, posing a significant new threat to public health. This variant is particularly notable due to the extensive number of mutations it has accumulated [23]. The most dominant strain currently is now the FLiRT variant, which is derived from the omicron lineage. FLiRT variants, including subvariants such as KP.3, KP.2, and KP.1.1, account for a significant portion of new cases [24, 25]. These variants are particularly notable for their enhanced ability to evade immunity and diagnostic detection, whether from previous infections or vaccinations as a result of specific mutations in the spike protein, that increase their transmissibility and ability to bypass immune defences [24, 25]. Furthermore, the symptoms of FLiRT infections remain similar to earlier variants, including sore throat, body aches, cough, runny nose, and shortness of breath, particularly in unvaccinated individuals or those whose immunity has waned over time [24]. The emergence of these variants could potentially lead to a surge in cases, as vaccines are ineffective against these variants [24].

In response, research institutions and pharmaceutical companies rapidly developed drugs and vaccines that could effectively target SARS-CoV-2 variants [26]. Thus, drugs such as oseltamivir, lopinavir, ritonavir, remdesivir, favipiravir, ribavirin, and chloroquine demonstrated some potency with the first cases of COVID-19 but they were proven to be ineffective in immunocompromised patients and those with comorbidities [27]. Up to this point, only a few therapeutic agents have been verified to exhibit certain levels of efficacy against SARS-CoV-2 strains of concern with the majority targeting one of the three key proteins associated with coronaviral pathogenicity [28]. While a handful have received approval, others are in the pipeline for official authorization, and some are still undergoing thorough investigation [28–32]. These agents encompass both synthetic and natural compounds [33], as well as newly developed [34, 35] or repurposed ones [36–38]. The most significant compounds, including engineered antibodies with an anticipated nonspecific efficacy against all emerging strains and potential future coronavirus species, are lengthily outlined in a scientific report [28]. Several vaccines received approval from the US Food and Drug Administration (US FDA), and even though they have been rolled out globally, they became less effective as new variants continued to emerge [39–42].

Natural products from medicinal plants, such as *Lycoris radiata*, *Rheum officinale, Curcuma longa*, and *Polygonum multiflorum* (just to name a few), have demonstrated some potency against various coronavirus strains [33, 43–45]. *I. cylindrica* also displays interesting characteristics that highlight its potential in the development of therapeutic compounds against pathogens such as SARS-CoV-2. One method that is mostly used to combat COVID-19 is by designing compounds that can hinder viral attachment to the host cells. SARS-CoV-2 is a positive-sense single-stranded (++SS) RNA virus with a genome of 14 open reading frames (ORFs) of 30kb [46]. Coronaviruses have 2 long polyproteins (pp1a and pp1ab), nine accessory proteins, and four major structural proteins, namely, the spike (S), the membrane (M), the envelope (E), and the nucleocapsid (N) that are encoded by these ORFs. The structural proteins are potent virulence factors involved in the replication, transmission, and viral host-cell interaction processes [46]. The S glycoprotein promotes viral entry into the host cell through binding with the host angiotensin-converting enzyme 2 (ACE-2) receptor [46]. This highly conserved protein is the main target of anti-SARS-CoV-2 drugs that are currently developed and investigated. Similarly, the main protease (M^pro^ or 3CL^pro^) and the papain-like protease (PL^pro^) are located within the ORF1a that are highly conserved among the variants of SARS-CoV, and they constitute ideal targets of the current drug candidates since human proteases do not have similar cleavage patterns [46–48]. These are cysteine proteases that are involved in the cleavage and the maturation of the polyproteins pp1a and pp1ab into nonstructural proteins (NSPs), including RNA-dependent RNA polymerase (RdRp), helicase, exoribonucleases, 2′-O-methyltransferase, and uridine-specific endoribonuclease [47]. Thus far, SARS-CoV-2 M^pro^ inhibitors (Ensitrelvir, Simnotrelvir, and Nirmatrelvir) [49–51] and RdRp inhibitors (Remdesivir, Molnupiravir, Renmindevir, and Azvudine) [51–55] have been approved by the US FDA and the China Medical Products Administration but they present setbacks such as suboptimal potency, poor oral bioavailability, low oral drug exposure, moderate stability in human hepatic microsomes [53–55], and emergence of variants that are resistant to these drugs [56–62].

The aim of this study is to use molecular docking tools to screen potential *I. cylindrica* phytochemicals that display antiviral activities against SARS-CoV-2 through molecular docking assays.

## 2. Materials and Methods

### 2.1. Protein Preparation

As highlighted in the literature, the main protease (M^pro^) is an essential protein in the replication of SARS-CoV-2 and it is highly conserved among the different variants [47]. It is also one of the main targets of the current antivirals that are developed against SARS-CoV-2 [48]. Thus, the three-dimensional X-ray crystal structure of the SARS-CoV-2 main protease with the identifier PDB ID: 6LU7 (resolution: 2.16 Å) was retrieved from the Research Collaboratory Structural Bioinformatics-Protein Data Bank (RCSB-PDB) and was used for *in sili*co studies. It was first visualised on BIOVIA Discovery Studio® version 4.0 modelling environment (Dassault Systèmes, San Diego, 2021) [63]. Water molecules and heteroatoms were then removed, and polar hydrogen was added to all noncarbon atoms to fill valencies. Finally, the restrained energy of the structure was minimised as previously described [64].

### 2.2. Ligand Preparation

Seventy-two *I. cylindrica* phytochemicals were selected for this assay. The N3 inhibitor (Supplementary Figure 1) was used to validate the procedure used in this study. The library was prepared manually by downloading structure data file (SDF) formats of flavonoids, saponins, phenols, coumarins, glycosides, and other unclassified phytochemicals of *I. cylindrica* from PubChem database (https://pubchem.ncbi.nlm.nih.gov/) as well as the PDB format of the N3 inhibitor. Their drug likeness was assessed as per Lipinski's rule of five [65]. The ligands were energy-minimised by inducing the universal force field (UFF) and converted into .pdbqt format with OpenBabel® (version 3.1.1x64) [66]. Hydroxychloroquine was used for comparative purposes in this assay, and its SDF file was retrieved from DrugBank database (accession number: DB01611) (https://go.drugbank.com/drugs/DB01611).

### 2.3. Binding Sites' Prediction and Receptor Grid Generation

The virtual screening and the molecular docking of the phytochemicals were carried out using Autodock Vina [67]. This turnkey software stands out as one of the swiftest and extensively employed open-source docking engines which relies on a straightforward scoring function and swift gradient-optimization conformational search. BIOVIA Discovery Studio® (version 4.0) was used to generate the receptor-binding grid. The grid box was large enough to cover the binding site of the protein structure (*x*, *y*, and *z* coordinates were −10.729204, 12.417653, and 68.816122, respectively). An exhaustiveness value of 9 was applied to maximise the probability of detecting global minimum scoring function in Autodock Vina. The interaction of the hit phytochemicals was assessed for their 2D and 3D interactions. Phytochemicals with the highest binding affinities were assessed with Discovery Studio Visualiser (version 4.0).

### 2.4. Pharmacokinetic Properties' Predictions

The SwissADME web tool (https://www.swissadme.ch/) [68] was used to predict the absorption, distribution, metabolism, and excretion (ADME) profiles of the phytochemicals that were druggable (as a result of a suitable molecular weight and a demonstrated binding energy). To predict the toxicity and the toxicological effects of the phytochemicals, ProTox-II web tool was used [69].

### 2.5. Molecular Dynamics Simulation

Molecular dynamics (MD) simulations of the most promising compound docked to M^pro^ was performed with the Desmond package. The complex was solvated in an explicit water box of size 10 Å using the TIP3P water model with periodic boundary conditions (PBC). The OPLS3e force field was employed to model the protein, ligand, and Na+/Cl− ions were assigned to neutralise the system's total charge. The system underwent energy minimisation for 2000 steps before a 60 ns production run. After minimization, the complex was subjected to a production run in the NPT ensemble. The system was gradually heated to maintain a temperature of 300 K and pressure using the Nose–Hoover thermostat algorithm and the Martina–Tobias–Klein method. Long-range electrostatic interactions were calculated using the particle mesh Ewald (PME) method with a grid spacing of 0.8 Å. Final trajectories were analyzed using UCSF Chimera [70]. The ligand-binding energy using MM-PBSA was calculated for the analyses. The molecular mechanics potential energy and the free energy of solvation for each complex were analyzed using the equation Δ*G*_binding_ = *E*_gas_ + *G*_sol_ − *T*Δ*S* considering all frames from the MD simulation trajectories [71].

## 3. Results

### 3.1. Molecular Docking Results

Of the seventy-two *I. cylindrica* phytochemicals that were docked, only eight phytochemicals displayed adequate binding affinities to SARS-CoV-2 main protease (Table 1). These phytochemicals included three compounds (bifendate, cylindrene, and tabanone) from the seven *I. cylindrica* unclassified phytochemicals, one coumarin (siderin), and four flavonoids (5-hydroxyl-2-[2-(2-hydroxyphenyl) ethyl]-4H-1-benzopyran-4-one, maritimin, 5-methoxyflavone, and flavone) (Table 1).

All the phytochemical binding affinities were higher than that of hydroxychloroquine and N3 inhibitor. Bifendate displayed the highest binding affinity (−9.1 kcal/mol) followed by 5-methoxyflavone (−7.2 kcal/mol), flavone (−7.1 kcal/mol), 5-hydroxyl-2-[2-(2-hydroxyphenyl) ethyl]-4H-1-benzopyran-4-one (−6.9 kcal/mol), cylindrene (−6.5 kcal/mol), siderin (−5.9 kcal/mol), maritimin (−5.9 kcal/mol), and tabanone (−5.6 kcal/mol), respectively (Table 1).

**Figure 2:**
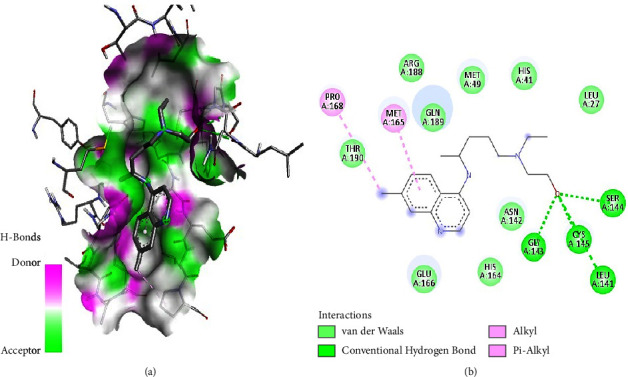
(a) Hydroxychloroquine best binding pose and binding site with M^pro^. (b) Hydroxychloroquine amino acid residues within the M^pro^ binding pocket.

**Figure 3:**
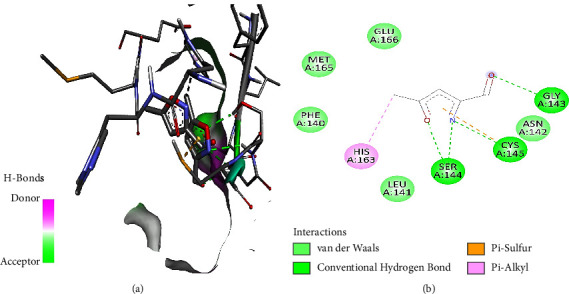
(a) N3 inhibitor best binding pose and binding site with M^pro^. (b) N3 inhibitor amino acid residues within the M^pro^ binding pocket.

As illustrated in the figures below, the best docking poses and the molecular interactions between the various docked phytochemicals and SARS-CoV-2 main protease (M^pro^) were recorded. Hydroxychloroquine and N3 inhibitor molecular interactions with SARS-CoV-2 main protease were also illustrated (Figures 2(a) and 3(a)). Hydroxychloroquine interacted with SARS-CoV-2 main protease-binding pocket by forming stable hydrogen bonds at amino acid residues Gly143, Leu141, Ser144, and Cys145. Alkyl bonds with M^pro^ were formed at amino acid residues Pro168 and Met165 (Figure 2(b)). The N3 inhibitor bonded with the SARS-CoV-2 M^pro^ binding pocket through the formation of stable hydrogen bonds at amino acid residues Gly143, Cys145, and Ser144. In addition, an alkyl bond and a Pi sulphur bond were also formed at the amino acids residues His163 and Cys145, respectively (Figure 3(b)).

**Figure 4:**
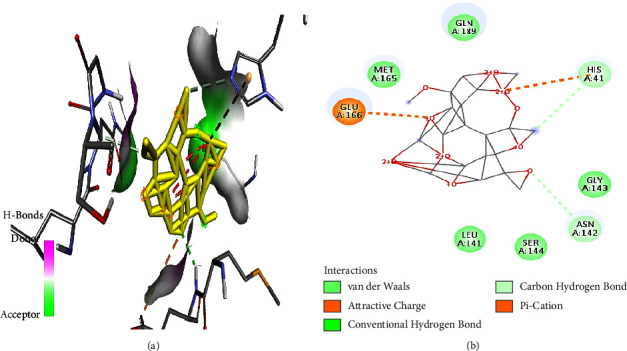
(a) Bifendate best binding pose and binding site with M^pro^. (b) Bifendate amino acid residues within the M^pro^ binding pocket.

Bifendate interacted with M^pro^ through cationic bonds (Figure 4(a)) at amino acid residues His41 and Glu166 as well as van der Waals bonds at amino acid residues His41 and Asn142 (Figure 4(b)).

**Figure 5:**
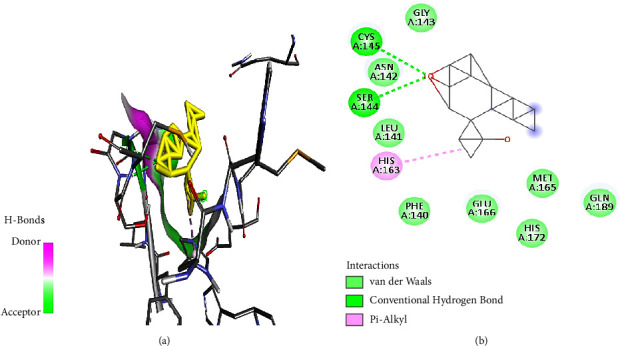
(a) Cylindrene best binding pose and binding site with M^pro^. (b) Cylindrene amino acid residues within the M^pro^ binding pocket.

Furthermore, cylindrene interacted with SARS-CoV-2 M^pro^ through hydrogen bonds (Figure 5(a)) at amino residues Cys145 and Ser144, and it also formed a Pi-alkyl bond at amino acid residue His163 (Figure 5(b)).

**Figure 6:**
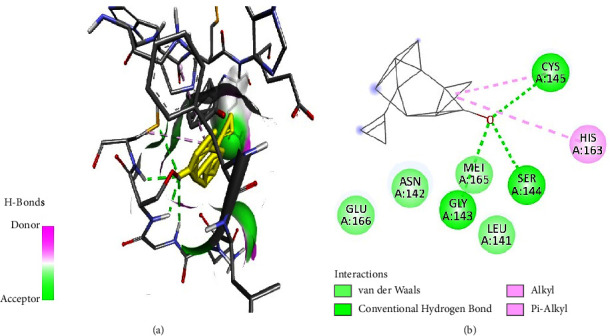
(a) Tabanone best binding pose and binding site with M^pro^. (b) Tabanone amino acid residues within the M^pro^ binding pocket.

**Figure 7:**
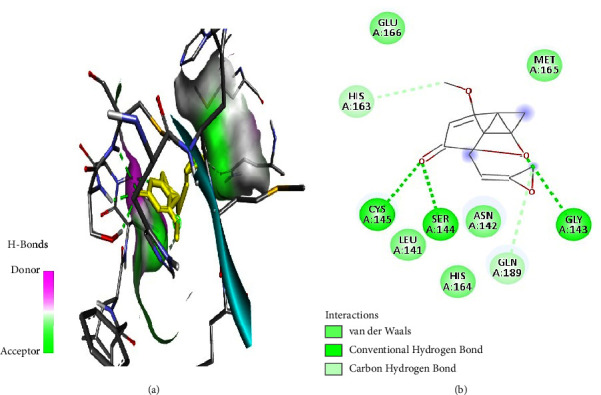
(a) Siderin best binding pose and binding site with M^pro^. (b) Siderin amino acid residues within the M^pro^ binding pocket.

**Figure 8:**
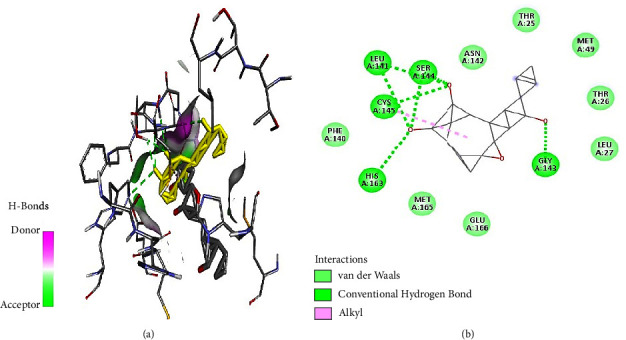
(a) 5-Hydroxy-2-[2-(2-hydroxyphenyl)ethyl]-4H-1-benzopyran-4-one best binding pose and binding site with M^pro^. (b) 5-Hydroxy-2-[2-(2-hydroxyphenyl)ethyl]-4H-1-benzopyran-4-one amino acid residues within the M^pro^ binding pocket.

**Figure 9:**
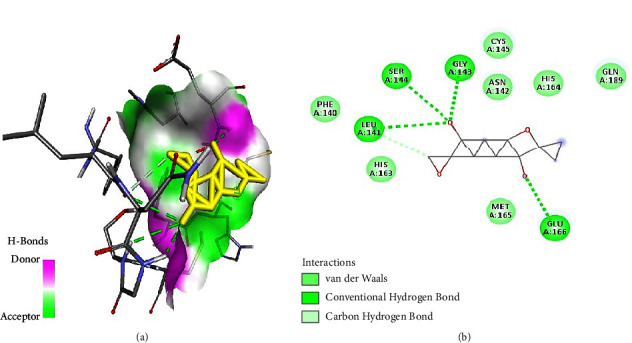
(a) Maritimin best binding pose and binding site with M^pro^. (b) Maritimin amino acid residues within the M^pro^ binding pocket.

**Figure 10:**
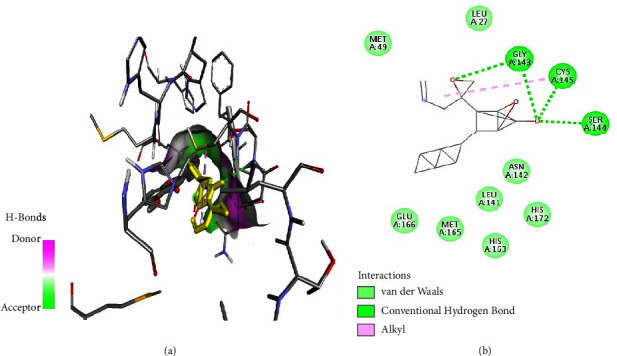
(a) 5-Methoxyflavone best binding pose and binding site with M^pro^. (b) 5-Methoxyflavone amino acid residues within the M^pro^ binding pocket.

**Figure 11:**
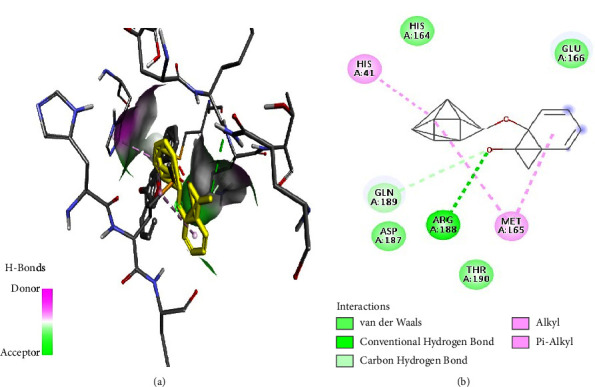
(a) Flavone best binding pose and binding site with M^pro^. (b) Flavone amino acid residues within the M^pro^ binding pocket.

Tabanone and siderin best binding poses highlighted an interaction between these ligands and SARS-CoV-2 M^pro^ through three hydrogen bonds at amino acid residues Cys145, Ser144, and Gly143 (Figures 6(b) and 7(b)). Tabanone also formed alkyl bonds at His163 and Cys145 (Figure 6(b)) while siderin displayed van der Waals interactions at residues Gln189 and His163 (Figure 7(b)). 5-Hydroxy-2-[2-(2-hydroxyphenyl)ethyl]-4H-1-benzopyran-4-one best binding pose with SARS-CoV-2 M^pro^ was formed through hydrogen bonds at amino acid residues Gly143, His163, Cys145, Ser144, and Leu141 (Figure 8(b)). Maritimin interacted with M^pro^ at amino acid residues Ser144, Gly143, Leu141, and Glu166 (Figure 9(b)) while 5-methoxyflavone interacted with M^pro^ through hydrogen bonds at amino acid residues Gly143, Cys145, and Ser144 and a Pi-alkyl bond at Cys145 (Figure 10(b)). Finally, flavone interacted with M^pro^ through a hydrogen bond at amino acid residue Arg188, a van der Waals bond at amino residues Gln189 and alkyl bonds at amino residues Met165 and His41 (Figure 11(b)).

### 3.2. Drug Likeness and Pharmacokinetic Properties

**Figure 12:**
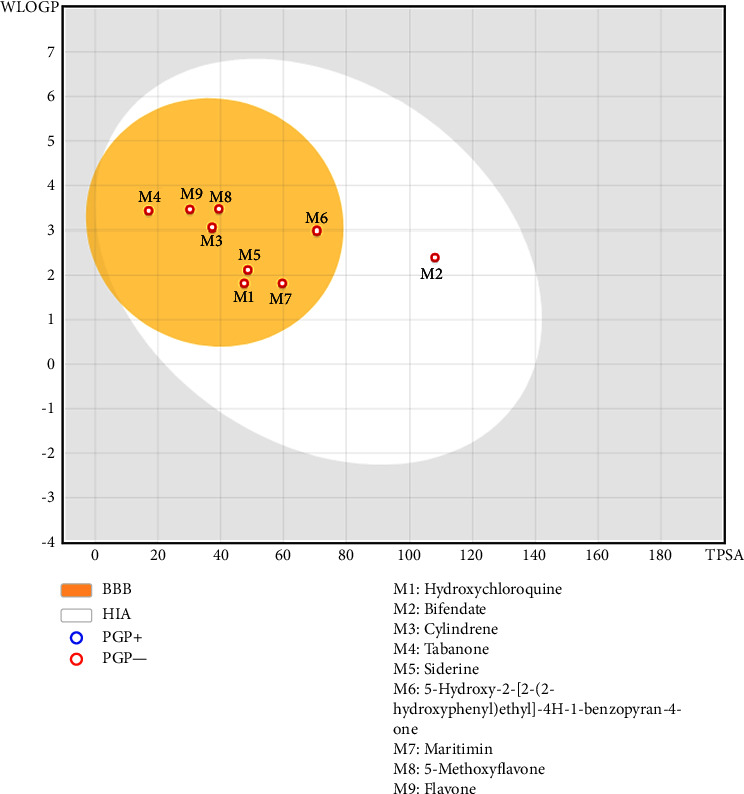
Egg diagram of *I. cylindrica* phytochemicals with the best docking scores and hydroxychloroquine. WLOGP: lipophilicity; TPSA: topological polar surface area; BBB: blood-brain barrier; HIA: high intestinal absorption; PGP: P-glycoprotein.

All the screened phytochemicals had zero Lipinski, Veber, Muegge, Ghose, and Egan violations (Table 2); a bioavailability score of 0.55; and a high gastrointestinal absorption index (Table 3). In addition, bifendate, siderin, 5-hydroxy-2-[2-(2-hydroxyphenyl) ethyl]-4H-1-benzopyran-4-one, maritimin, 5-methoxyflavone, and flavone were observed as inhibitors of cytochrome P1A2 (CYP1A2) while cylindrene and tabanone were not (Table 3). Furthermore, cylindrene, tabanone, siderin, and maritimin do not inhibit CYP2C19 while the other phytochemicals do. Similarly, CYP2C9 is inhibited by bifendate, 5-hydroxy-2-[2-(2-hydroxyphenyl) ethyl]-4H-1-benzopyran-4-one, and 5-methoxyflavone. In addition, cylindrene, 5-hydroxy-2-[2-(2-hydroxyphenyl)ethyl]-4H-1-benzopyran-4-one and 5-methoxyflavone are the only CYP2D6 inhibitors, while bifendate, 5-hydroxy-2-[2-(2-hydroxyphenyl)ethyl]-4H-1-benzopyran-4-one, and 5-methoxyflavone are the only CYP3A4 inhibitors (Table 3). The only phytochemical that was not permeable through the blood brain barrier was bifendate but all the phytochemicals displayed a high intestinal absorption (Table 3 and Figure 12).

The prediction of permeability coefficient (Kp) for the transport of compounds through mammalian epidermis is based on the linear model by Potts and Guy and indicates the ability of a compound to pass through the mammalian skin. The more negative the log Kp value of a molecule, the less skin-permeant that molecule will be [72]. 5-hydroxy-2-[2-(2-hydroxyphenyl)ethyl]-4H-1-benzopyran-4-one and 5-methoxyflavone displayed the highest capacity of skin permeability (−4.8 and −4.94, respectively) as compared to bifendate which displayed a poor capacity to permeate the mammalian skin (−6.85) (Table 3). All the investigated phytocompounds displayed a high gastrointestinal absorption index and a good bioavailability score but only bifendate was not permeant to the blood brain barrier (BBB) (Table 3). P-glycoprotein, an ABC transporter that has undergone extensive research, acts as a biological barrier by expelling toxins and xenobiotics from cells. Both laboratory experiments and studies involving living organisms have shown that P-glycoprotein significantly influences the absorption and distribution of drugs [73]. In theory, Pgp-inducers (or Pgp substrates) can cause a reduction in the drug bioavailability, an increased renal clearance of the drug and a reduced distribution in the peripheral tissues [74]. Therefore, an ideal drug candidate should not be a Pgp substrate. All the phytocompounds investigated in this report are not Pgp substrate (Table 3).

### 3.3. Toxicity Assessment Results

The toxicity prediction outcomes indicated that none of the phytochemicals has the tendency to be hepatotoxic (Table 4). However, maritimin tended to be carcinogenic and highly mutagenic while flavone displayed the tendency to be carcinogenic and highly cytotoxic. 5-methoxyflavone displayed the tendency to be fairly carcinogenic, fairly mutagenic, and fairly cytotoxic (Table 4). The median lethal dose (or LD50) is defined as the dose of a test substance that is lethal for 50% of the animals in a dose group [75]. The most lethal phytochemical was observed to be maritimin (100 mg/kg) followed by bifendate (777 mg/kg), 5-hydroxy-2-[2-(2-hydroxyphenyl)ethyl]-4H-1-benzopyran-4-one (832 mg/kg), cylindrene (1700 mg/kg), flavone (2500 mg/kg), siderin (2850 mg/kg), 5-methoxyflavone (4000 mg/kg), and tabanone (10000 mg/kg), respectively (Table 4).

### 3.4. Molecular Dynamics Simulation

**Figure 13:**
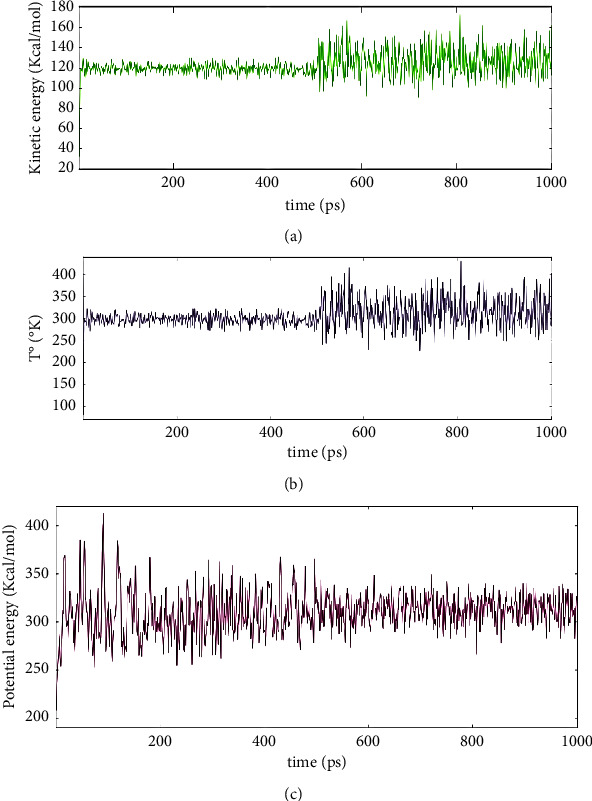
Tabanon/6LU7 complex kinetic energy plot (a); temperature plot (b); atomic potential energy function (c).

The conformational flexibilities of the tabanone-docked compound with the M^pro^ enzyme (PDB: 6LU7) were examined via MD simulations to obtain reliable drug-receptor-binding affinities.  Figure 13 displays its kinetic energy plot (A), its stability on a temperature versus time plot (B), and its atomic potential energy function (C). As shown in Figure 13(a), the complex kinetic energy remains stable until around 500 ps. Its remains stable at temperatures varying from 260 to 350 K until around 500 ps (Figure 13(b)). To assess the dynamic stability of the 6LU7/tabanone complex, its time-dependent potential energy was calculated throughout the MD trajectory and its potential energy stabilises at around 500 ps (Figure 13(c)).

## 4. Discussion

SARS-CoV-2 variants of concern (VOCs) such as omicron and its sublineages are highly transmissible and still constitute a source of concern [40]. Despite vaccinations, breakthrough infections, hospitalizations, and mortality rates resulting from infections by these VOCs are still high and existing therapeutic alternatives have presented various setbacks such as low potency, poor pharmacokinetic profile, and drug resistance [26, 58, 60, 61]. Even though there are promising therapeutic options that could alleviate the burden of symptoms resulting from infection by SARS-CoV-2 VOCs [28, 29, 32, 34–38, 76–79], there is still a need to identify compounds that could be effective against these variants, especially in this context of emerging resistant strains. Phytochemicals are still poorly investigated, they display interesting properties that make them ideal substitutes in the design and the development of novel therapeutic compounds, and *in silico* modelling of phytocompounds has yield interesting results that could be highly relevant in drug design and development [80–82]. Natural compounds had proven to be effective on SARS-CoV 2 by previous studies [33, 43–45, 79]. In this study, we used molecular docking tools to assess the antiviral properties of *Imperata cylindrica* phytochemicals against SARS-CoV-2 and their overall safety. Of the 72 compounds that are commonly found in this plant, eight displayed interesting docking results against SARS-CoV-2 main protease (M^pro^), a highly conserved protein among all the coronaviruses [47]. M^pro^ is a homodimer which is vital in viral replication and the maturation of coronavirus nonstructural proteins, and it is an ideal drug target as humans do not possess its homologue. It is structured by three domains which configure it into two substrate-binding sites: an oxyanion hole (which is made up of Gly143, Ser144, and Cys145) and a substrate-binding pocket [83]. The substrate-binding pocket includes su-sites s1 (F140, L141, N142, H163, and E166), s2 (M49, Y54, H164, D187, and R188), s4 which is hydrophobic (M165, L167, Q189, T190, and Q192), and hydrophilic subsites such as s3 (E166), s5 (including T190, A191, and Q192), and s1′ (H41, G143, S144, and C145) [84–86]. The s1′ subsite is a catalytic dyad with residues Cys145-His41 where cysteine acts as a nucleophile while histidine plays the role of a proton acceptor. M^pro^ inhibitors act through various modes of action. One of the mechanisms is by forming covalent bonds with Cys145-His41 of the catalytic dyad, leading to M^pro^ inhibition. Another mode of action is by embedding in the oxyanion hole of M^pro^ [86].

Bifendate displayed the highest binding affinity (−9.1 kcal/mol) to M^pro^ and would be safe for human consumption based on the toxicological assessment data. It may be effective in inhibiting the main protease because it bound to the protein by forming van der Waal bonds with amino acid residues Asn142 and His41, and Pi-cationic bonds with amino acid residues Glu166 and His41. However, it would be a poor antiviral drug candidate due to the following: (i) no hydrogen bonds were formed with the M^pro^ binding sites, (ii) it is not permeant to the blood brain barrier, and (iii) it inhibits CYP1A2, CYP2C19, CYP2C9, and CYP3A4. The binding affinity of cylindrene was adequate (−6.5 kcal/mol) and it interacted with SARS-CoV-2 M^pro^ by forming hydrogen bonds at amino residues Cys145 and Ser144 of the oxyanion hole, and a Pi-alkyl bond at amino acid residue His163. These parameters coupled with its acceptable pharmacokinetic profile and safety (based on toxicological assessment) suggest that cylindrene would be one of the best drug candidates to inhibit M^pro^. Tabanone and siderin also formed stable hydrogen bonds with M^pro^ oxyanion hole at amino acid residues Cys145, Ser144, and Gly143. In addition, tabanone formed two alkyl bonds at amino acid residues His163 and Cys145, while siderin formed van der Waals bonds at amino acid residues His163 and Gln189. These two phytochemicals can therefore inhibit M^pro^. However, siderin would be a poor drug candidate despite its acceptable pharmacokinetic properties. As revealed in this study, siderin is carcinogenic and mutagenic when compared to tabanone which is safer with a predicted LD50 of 10,000 mg/kg (meaning that it would be lethal only at high doses). Interestingly, 5-hydroxy-2-[2-(2-hydroxyphenyl)ethyl]-4H-1-benzopyran-4-one formed six covalent hydrogen bonds in the M^pro^ oxyanion hole by binding to Ser144, Cys145, His163, Gly143, and Leu141 amino acid residues, respectively. Besides the fact that it inhibits CYP1A2, CYP2C19, CYP2C9, CYP2D6, and CYP3A4, its other pharmacokinetic parameters are relatively acceptable and it is safe for human consumption. Contrastingly, even though maritimin, 5-methoxyflavone, and flavone formed stable hydrogen bonds with amino acid residues of the M^pro^ oxyanion-binding site, they are unsafe. In particular, maritimin is carcinogenic and highly mutagenic with an intriguing lethal dose of 100 mg/kg. Flavone is carcinogenic and highly cytotoxic while 5-methoxyflavone may be carcinogenic, mutagenic, and cytotoxic. These features indicate that these phytochemicals are not acceptable drug candidates. The findings on these *I. cylindrica* flavonoids (5-methoxyflavone and flavone) are contrasting with another study which demonstrated the inhibitory properties of 31 polyphenolic and flavonoid bioactive compounds on SARS-CoV-2 main protease and papain-like protease (PLpro) enzymes [87]. This indicates that the binding of bioactive compounds can undergo structural adjustments in such a way that they interfere with the enzyme functions and this can be achieved through molecular dynamics simulation [87].

Conclusively, tabanone seems to be the best candidate for the development of an M^pro^ inhibitor, among all the *I. cylindrica* phytochemicals tested in this study. This was justified by the following observations: (i) tabanone will be lethal to human beings only at very high doses (predicted LD50 = 10000 mg/kg) (Table 4 and Supplementary Table 2); (ii) it is neither hepatotoxic nor cytotoxic (Table 4); (iii) it is neither carcinogenic nor immunotoxic (Table 4); (iv) it is not mutagenic (Table 4) and it displayed a good skin permeability index (−5.61 cm/S) (Table 3); (v) its bioavailability score, its gastrointestinal absorption index, and its intestinal absorption index are high (Table 3); (vi) it displayed a high ability to pass through the blood brain barrier; (vii) it is not a P-glycoprotein substrate and it does not inhibit any of the cytochromes (Table 3 and Supplementary Table 2); (viii) it neither violated any of Lipinski's rule of five nor that of the other scientists (Veber, Muegge, Ghose, and Egan) (Table 3 and Supplementary Table 2). As compared to hydroxychloroquine (binding affinity = −5.4) and N3 inhibitor (binding affinity = −3.7), tabanone displayed a better binding affinity to SARS-CoV-2 M^pro^ (binding affinity = −5.6) (Table 1). They all interacted with the active site of the main protease-binding pocket by forming covalent and stable hydrogen bonds at amino acid residues Gly143, Ser144, and Cys145 (Figures 2(b), 3(b), and 6(b)). However, only N3 inhibitor and tabanone were able to form an alkyl bond with M^pro^ at amino acid residue His163. Furthermore, the MD simulations indicate that tabanone is stable when forming a complex with the M^pro^ enzyme (Figures 13(a), 13(b), and 13(c)).

This study is the first to assess *I. cylindrica* phytochemicals against a viral protein using molecular docking assays. However, a similar study investigated the inhibitory properties of phytocompounds from four medicinal plants (*Salvia officinalis* L, *Anacardium occidentale*, *Crinum jagus*, and *Andrographis paniculata*) against SARS-CoV nsp16 protein (a conserved protein that is involved in coronavirus RNA methylation) [88]. Of the 100 phytocompounds that were screened, 59 passed the drug likeness assays and only six compounds (oxoproline, andrographolide, deacetylbowdensine, 12-dimethyl sageone, sageone, and quercetin) showed good binding affinities after the docking process, with docking scores that ranged from −7.9 to −8.4 kcal/mol. The toxicological profiles of all the six compounds were acceptable, making the compounds ideal candidates for the development of nsp16 inhibitors. Furthermore, another study investigated the inhibitory properties of *Andrographis paniculata* (a plant that is used as an anti-inflammatory and an antiviral therapy) phytochemicals and the nitrobenzoxadiazole-conjugated andrographolide (andro-NBD) suppressed SARS-CoV M^pro^ activities [89]. In addition, *Zanthoxylum piperitum*, *Withania somnifera*, *Calophyllum inophyllum*, and *Centella asiatica* phytochemicals were found to be effective against SARS-CoV-2 after *in silico* screening (with molecular docking), *in vitro* and *in vivo* assays [90]. Another study docked the bioactive phytocompounds of tea against SARS-CoV-2 nonstructural protein 15 (nsp15) [91]. Three molecules from the tea extract (barrigenol, kaempferol, and myricetin) displayed adequate docking scores. Similarly, another interesting study investigated the inhibitory potential of an ayurvedic herbal formulation (*Triphala*) on SARS-Cov-2 main protease, and it was demonstrated that some of its phytocompounds (terflavin A, chebulagic acid, chebulinic acid, and corilagin) where potential inhibitors of M^pro^. In summary, several other studies have investigated the inhibitory effects of phytocompounds on SARS-CoV-2 main protease and other vital proteins (such as PLpro and RdRp) [92–95], and they reported acceptable docking scores showing that medicinal plants constitute potential alternatives in the development of therapeutic drugs against SARS-CoV VOCs [96–105].

Further *in vitro* and *in vivo* assessment of these phytochemicals against SARS-CoV-2 proteins should be considered to validate the findings of this study.

## 5. Conclusion

The current antiviral agents that are destined to cure COVID-19 through the inhibition of coronavirus main protease, a highly conserved protein among coronaviruses, have demonstrated limitations such as the rise of resistant strains to these drugs, poor pharmacokinetic profiles, and a suboptimal potency, rendering them ineffective against emerging strains of SARS-CoV-2. This calls for the development of new therapeutic options that could be effective against such variants of concern. Phytochemicals of medicinal plants present as unexplored potentials which makes them ideal candidates for the development of effective therapeutic alternatives. *Imperata cylindrica* phytochemicals were screened with molecular docking tools and *in silico* pharmacokinetic assays in this study. Of the 79 phytocompounds that were investigated, bifendate, cylindrene, tabanone, siderin, 5-hydroxy-2-[2-(2-hydroxyphenyl)ethyl]-4H-1-benzopyran-4-one, maritimin, 5-methoxyflavone, and flavone displayed the best docking scores. Tabanone was revealed as the safest compound after toxicological assessment and the best drug candidate based on its pharmacokinetic profile. Tabanone could be used in the development of an efficient M^pro^ inhibitor, provided that adequate *in vitro* and *in vivo* assays are conducted to support the findings of this study.

## Figures and Tables

**Table 1:** *Imperata cylindrica* phytochemicals with the best docking results.

Phytochemicals		Binding affinity (kcal/mol)	Distance from best mode	
			RMSD 1.b	RMSD U.b
Unclassified compounds	Bifendate	−9.1	3.158	5.582
	Cylindrene	−6.5	5.095	7.157
	Tabanone	−5.6	5.245	6.894

Coumarins	Siderin	−5.9	4.298	6.221

Flavonoids	5-Hydroxy-2-[2-(2-hydroxyphenyl)ethyl]-4H-1-benzopyran-4-one	−6.9	7.913	10.129
	Maritimin	−5.9	4.281	5.739
	5-Methoxyflavone	−7.2	6.065	7.911
	Flavone	−7.1	1.105	4.055

Control	Hydroxychloroquine	−5.4	3.700	6.489

Validation	N3 inhibitor	−3.7	7.569	7.804

**Table 2:** Drug likeness of *I. cylindrica* phytochemicals with the best docking scores.

Phytochemicals	Formula	MW	RB	HBA	HBD	TPSA	MR	iLOGP	Lipinski violations	Veber violations	Muegge violations	Ghose violations	Egan violations
Hydroxychloroquine	C18H36ClN3O	345.95	9	4	3	47.53	102.78	3.76	0	0	0	0	0
Bifendate	C20H18O10	418.35	7	10	0	107.98	99.55	3.54	0	0	0	0	0
Cylindrene	C15H20O2	232.32	2	2	1	37.3	69.69	2.8	0	0	0	0	0
Tabanone	C13H18O	190.28	1	1	0	17.07	61.01	2.72	0	0	0	0	0
Siderin	C12H12O4	220.22	2	4	0	48.67	60.43	2.58	0	0	0	0	0
5-Hydroxy-2-[2-(2-hydroxyphenyl)ethyl]-4H-1-benzopyran-4-one	C17H14O4	282.29	3	4	2	70.67	80.79	2.61	0	0	0	0	0
Maritimin	C11H10O4	206.19	1	4	1	59.67	55.97	1.78	0	0	0	0	0
5-Methoxyflavone	C16H12O3	252.26	2	3	0	39.44	74.41	2.65	0	0	0	0	0
Flavone	C15H10O2	222.24	1	2	0	30.21	67.92	2.55	0	0	0	0	0

MW: molecular weight; RB: rotatable hydrogen bonds; HBA: hydrogen bond acceptors; HBD: hydrogen bond donors; TPSA: topological polar surface area; MR: molar reactivity; iLOGP: octanol-water partition coefficient.

**Table 3:** Pharmacokinetic properties of *I. cylindrica* phytochemicals with the best docking scores.

Phytochemicals	Formula	MW	Log Kp (cm/s)	BS	GIA	BBB permeant	Pgp substrate	CYP1A2 inhibitor	CYP2C19 inhibitor	CYP2C9 inhibitor	CYP2D6 inhibitor	CYP3A4 inhibitor
Hydroxychloroquine	C18H36ClN3O	345.95	−6.81	0.55	High	Yes	No	No	No	No	Yes	No
Bifendate	C20H18O10	418.35	−6.85	0.55	High	No	No	Yes	Yes	Yes	No	Yes
Cylindrene	C15H20O2	232.32	−5.97	0.55	High	Yes	No	No	No	No	Yes	No
Tabanone	C13H18O	190.28	−5.61	0.55	High	Yes	No	No	No	No	No	No
Siderin	C12H12O4	220.22	−6.27	0.55	High	Yes	No	Yes	No	No	No	No
5-Hydroxy-2-[2-(2-hydroxyphenyl)ethyl]-4H-1-benzopyran-4-one	C17H14O4	282.29	−4.8	0.55	High	Yes	No	Yes	Yes	Yes	Yes	Yes
Maritimin	C11H10O4	206.19	−6.12	0.55	High	Yes	No	Yes	No	No	No	No
5-Methoxyflavone	C16H12O3	252.26	−4.94	0.55	High	Yes	No	Yes	Yes	Yes	Yes	Yes
Flavone	C15H10O2	222.24	−5.13	0.55	High	Yes	No	Yes	Yes	No	No	No

MW: molecular weight; log Kp: skin permeation; BS: bioavailability score; GIA: gastrointestinal absorption; BBB: blood-brain barrier; Pgp: P-glycoprotein; CYP1A2: cytochrome P1A2; CYP2C19: cytochrome P2C19; CYP2C9: cytochrome P2C9.

**Table 4:** Toxicity assessment of *I. cylindrica* phytochemicals with the best docking scores.

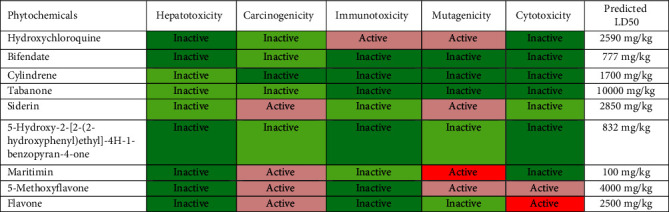

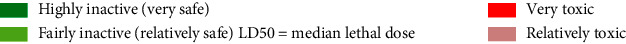

## Data Availability

The data used to support the findings of this study are available from the corresponding authors upon reasonable request.

## Supplementary Materials

Supplementary Materials Supplementary Table 1: data of N3 inhibitor docking on Mpro. Supplementary Figure 1: chemical structure of N3 inhibitor.
