# Supplementary material for: Predictive Assessment of the Antiviral Properties of Imperata cylindrica against SARS-CoV-2
Source: Adv Virol. 2024 Aug 4;2024:8598708. doi: 10.1155/2024/8598708 (PMC11317227; doi:10.1155/2024/8598708)
Supplement: Supplementary Materials — Supplementary Table 1: data of N3 inhibitor docking on Mpro. Supplementary Figure 1: chemical structure of N3 inhibitor. [file 8598708.f1.zip › Supplementary table 1 (1).docx]

**Supplementary table 1**: Data of N3 inhibitor docking on M^pro^

| **Mode (Pose)** | **Binding affinity (Kcal/mol)** | **Distance from best mode** | |
| --- | --- | --- | --- |
|  |  | **RMSD 1.b** | **RMSD U.b** |
| 1 | -4.4 | 0.000 | 0.000 |
| 2 | -4.0 | 1.321 | 3.678 |
| 3 | -4.0 | 7.497 | 8.037 |
| 4 | -3.7 | 6.919 | 7.646 |
| 5 | -3.7 | 7.569 | 7.804 |
| 6 | -3.6 | 3.237 | 3.712 |
| 7 | -3.5 | 7.222 | 7.443 |
| 8 | -3.3 | 2.682 | 2.906 |
| 9 | -3.3 | 5.396 | 6.757 |

**Supplementary table 2:** ADMET properties of tabanone

| Formula | MW | #Heavy atoms | #Aromatic heavy atoms | Fraction Csp3 | #Rotatable bonds | #H-bond acceptors | #H-bond donors | MR | TPSA | iLOGP | XLOGP3 | WLOGP |
| --- | --- | --- | --- | --- | --- | --- | --- | --- | --- | --- | --- | --- |
| C13H18O | 190.28 | 14 | 0 | 0.46 | 1 | 1 | 0 | 61.01 | 17.07 | 2.72 | 2.6 | 3.43 |

| MLOGP | Silicos-IT Log P | Consensus Log P | ESOL Log S | ESOL Solubility (mg/ml) | ESOL Solubility (mol/l) | ESOL Class | Ali Log S | Ali Solubility (mg/ml) | Ali Solubility (mol/l) | Ali Class | Silicos-IT LogSw | Silicos-IT Solubility (mg/ml) |
| --- | --- | --- | --- | --- | --- | --- | --- | --- | --- | --- | --- | --- |
| 2.85 | 3.66 | 3.05 | -2.59 | 4.87E-01 | 2.56E-03 | Soluble | -2.61 | 4.70E-01 | 2.47E-03 | Soluble | -2.85 | 2.71E-01 |

| Silicos-IT Solubility (mol/l) | Silicos-IT class | GI absorption | BBB permeant | Pgp substrate | CYP1A2 inhibitor | CYP2C19 inhibitor | CYP2C9 inhibitor | CYP2D6 inhibitor | CYP3A4 inhibitor | log Kp (cm/s) | Lipinski #violations | Ghose #violations |
| --- | --- | --- | --- | --- | --- | --- | --- | --- | --- | --- | --- | --- |
| 1.42E-03 | Soluble | High | Yes | No | No | No | No | No | No | -5.61 | 0 | 0 |

| Veber #violations | Egan #violations | Muegge #violations | Bioavailability Score | PAINS #alerts | Brenk #alerts | Leadlikeness #violations | Synthetic Accessibility |
| --- | --- | --- | --- | --- | --- | --- | --- |
| 0 | 0 | 2 | 0.55 | 0 | 0 | 1 | 3.58 |
