# Supplementary figures and images for: Predictive Assessment of the Antiviral Properties of Imperata cylindrica against SARS-CoV-2
Source: Adv Virol. 2024 Aug 4;2024:8598708. doi: 10.1155/2024/8598708 (PMC11317227; doi:10.1155/2024/8598708)

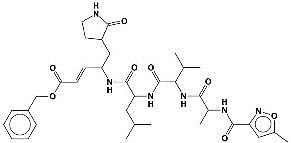


**Supplementay figure 1**: Chemical structure of N3 inhibitor

Supplement: Supplementary Materials — Supplementary Table 1: data of N3 inhibitor docking on Mpro. Supplementary Figure 1: chemical structure of N3 inhibitor. [file 8598708.f1.zip › Supplementay figure 1 (1).docx]
